# Updates on Laparoscopy Versus Laparotomy in the Management of Penetrating Abdominal Trauma: A Systematic Review

**DOI:** 10.7759/cureus.79231

**Published:** 2025-02-18

**Authors:** Yousef Alalawi, Nawaf Alharthi, Sultan Abdulrahman S Alamrani

**Affiliations:** 1 Department of Surgery, King Salman Armed Forces Hospital, Tabuk, SAU

**Keywords:** emergency, laparoscopy, laparotomy, penetrating abdominal trauma, systematic review, trauma

## Abstract

The purpose of this review is to contrast laparotomy with laparoscopy for penetrating abdominal trauma (PAT) in terms of efficacy, safety, and patient outcomes. A thorough search across four databases identified 416 relevant publications. After removing duplicates using Rayyan Qatar Computing Research Institute (QCRI) and screening for relevance, 36 full-text articles were reviewed, with five studies ultimately meeting the criteria for inclusion. There were 336 patients throughout five trials, with 273 (812%) of them being male. In total, 211 patients had laparoscopy, whereas 125 underwent laparotomy. From 2.9% to 17.9%, there was a conversion rate from laparoscopic to open approach. The review highlights that laparoscopy generally results in fewer complications compared to laparotomy, especially in stable individuals who have experienced piercing abdominal trauma. Laparoscopy is associated with shorter hospital stays, faster recovery, and fewer postoperative issues such as wound infections. However, in cases of retroperitoneal injuries or active bleeding, higher conversion rates to open surgery were observed. Despite these limitations, laparoscopy proves to be an effective and less invasive option for managing selected cases of abdominal trauma, reducing overall healthcare costs and postoperative morbidity. Laparoscopy offers a minimally invasive, practical choice for treating piercing abdominal injuries, especially in stable patients, with fewer postoperative complications and faster recovery compared to laparotomy. However, its limitations in managing more complex injuries warrant careful patient selection and readiness to convert to open surgery when necessary. It will take more investigation, especially randomized studies, to confirm laparoscopy's place in this sector.

## Introduction and background

In the first half of a person's life, trauma is the leading cause of mortality; however, in the general population, trauma is the fourth most prevalent cause of death [1]. Moreover, the abdomen is involved in 9%-14.9% of all trauma cases [2]. Abdominal trauma is a preventable cause of mortality for patients with polytrauma, and laparotomy has long been considered the gold standard of treatment for these patients [3,4]. However, unnecessary laparotomies should be avoided as they entail a morbidity rate of 20%-40% [5,6]. Laparoscopy, when performed by skilled surgeons under hemodynamically stable settings, is a safer and more efficient therapy for those suffering from abdominal trauma [7]. 

For those who have suffered abdominal injuries, exploratory laparotomy has been the standard of therapy. However, the morbidity and mortality of abdominal trauma have increased dramatically due to missed injuries, unnecessary laparotomies, and late diagnosis [8]. Although laparotomy is a dependable and effective method for treating individuals with abdominal injuries, the operation is risky; following the surgery, there is a 5% chance of mortality and a 3% long-term risk of intestinal blockage [9].

The first laparoscopy for abdominal trauma was performed in 1956 [10]. Laparoscopy is mostly used for diagnosis, with treatment being a secondary function [8]; however, a successful solution was achieved when therapeutic laparoscopy was used to identify and fix every injury. Laparoscopy is becoming increasingly common when identifying and treating abdominal trauma because of its potential benefits of minimizing unnecessary laparotomies, having a wide field of vision, being less invasive, and causing less discomfort [11,12].

The management of penetrating abdominal trauma (PAT) has evolved significantly with advancements in minimally invasive surgery. Traditionally, the gold standard for identifying and managing these injuries has been laparotomies, which are open surgical procedures [6]. However, with the growing expertise in laparoscopy, a less invasive alternative, there is a need to systematically evaluate its role in managing PAT. We conducted a systematic review to provide a thorough comparison of the relative efficacy, safety, and results of laparoscopy and laparotomy in the treatment of PAT [8].

Recent studies comparing laparoscopy and laparotomy for the management of PAT have highlighted both the advantages and limitations of these surgical techniques [5,6]. While laparoscopy offers benefits such as reduced postoperative pain, shorter hospital stays, and quicker recovery times, it is not always the most suitable option in trauma cases. One major limitation of laparoscopic approaches is the risk of hemodynamic instability, which frequently accompanies traumatic injuries. Patients in shock require rapid intervention and stabilization, and the open nature of laparotomy allows for faster access to major organs and blood vessels, providing an immediate opportunity to control hemorrhage and address life-threatening injuries. In contrast, the time-consuming setup required for laparoscopic procedures, including insufflation and multiple port placements, can be detrimental in urgent situations where every second counts [9,11].

Additionally, ongoing bleeding within the abdomen poses a significant challenge in laparoscopic trauma surgery. In cases of penetrating trauma, the presence of internal hemorrhage can obscure the surgical field, making it difficult for surgeons to identify and manage vital structures. This decreased visibility increases the risk of missing critical injuries or failing to achieve adequate hemostatic control. The slower and more methodical nature of laparoscopic exploration can further exacerbate issues of visualization, particularly in the dynamic setting of trauma where conditions can rapidly change [3]. As such, while there is a growing body of evidence supporting the use of laparoscopy in select cases of PAT, its limitations related to hemodynamic instability, ongoing bleeding, and slower operative tempos must be carefully considered in the decision-making process. In scenarios where immediate intervention is paramount, laparotomy often remains the preferred approach [11].

The objective was to critically assess current evidence, focusing on key outcomes such as morbidity, mortality, and complications. This review aims to guide clinical decision-making by summarizing the advantages and limitations of each approach, identifying gaps in the literature, and suggesting topics for further study.

## Review

Search strategy

The systematic review followed the Preferred Reporting Items for Systematic Reviews and Meta-Analyses (PRISMA) and Guidelines for Accurate and Transparent Health Estimates Reporting (GATHER) criteria. A thorough search was conducted on PubMed, Cochrane, Web of Science, and SCOPUS to find relevant studies regarding laparoscopy vs. laparotomy in PAT management. We considered only studies released between 2016 and 2024; after the computerized search, we removed any duplicates from the titles and abstracts entered into Rayyan [13]. All the studies that satisfied the inclusion criteria based on the title or abstract were then collected for comprehensive inspection. Two reviewers separately evaluated the appropriateness of the extracted papers and addressed any inconsistencies via discussion.

Study population selection

The population, intervention, comparison, and outcome (PICO) factors were implemented as inclusion criteria for our review: (i) population: patients with PAT; (ii) intervention: laparoscopy; (iii) comparator: laparotomy; and (iv) outcome: effectiveness and safety of the intervention. 

Data extraction

Data from studies that satisfied the inclusion criteria were collected in a standardized manner by two impartial reviewers. The following data were obtained and documented: (i) first author, (ii) year of publication, (iii) study design, (iv) study period, (v) participant number, (vi) participant age, (vii) participant gender, (viii) number of subjects in laparoscopy and laparotomy groups, (ix) conversion rate to open intervention, (x) complications, and (xi) main outcomes (efficacy, safety, and complications). 

Quality review

Because the "Risk Of Bias In Nonrandomized Studies-of Interventions" (ROBINS-I) approach allows for a thorough examination of confounding, we used it to evaluate the risk of bias, which is significant because bias owing to omitted variables is common in studies in this field. The ROBINS-I tool is intended to evaluate nonrandomized investigations and can be applied to cohort designs in which participants exposed to various staffing levels are monitored over time. Disagreements were settled through group discussions after each paper’s risk of bias was evaluated independently by two reviewers [14]. 

Results

**Figure 1:**
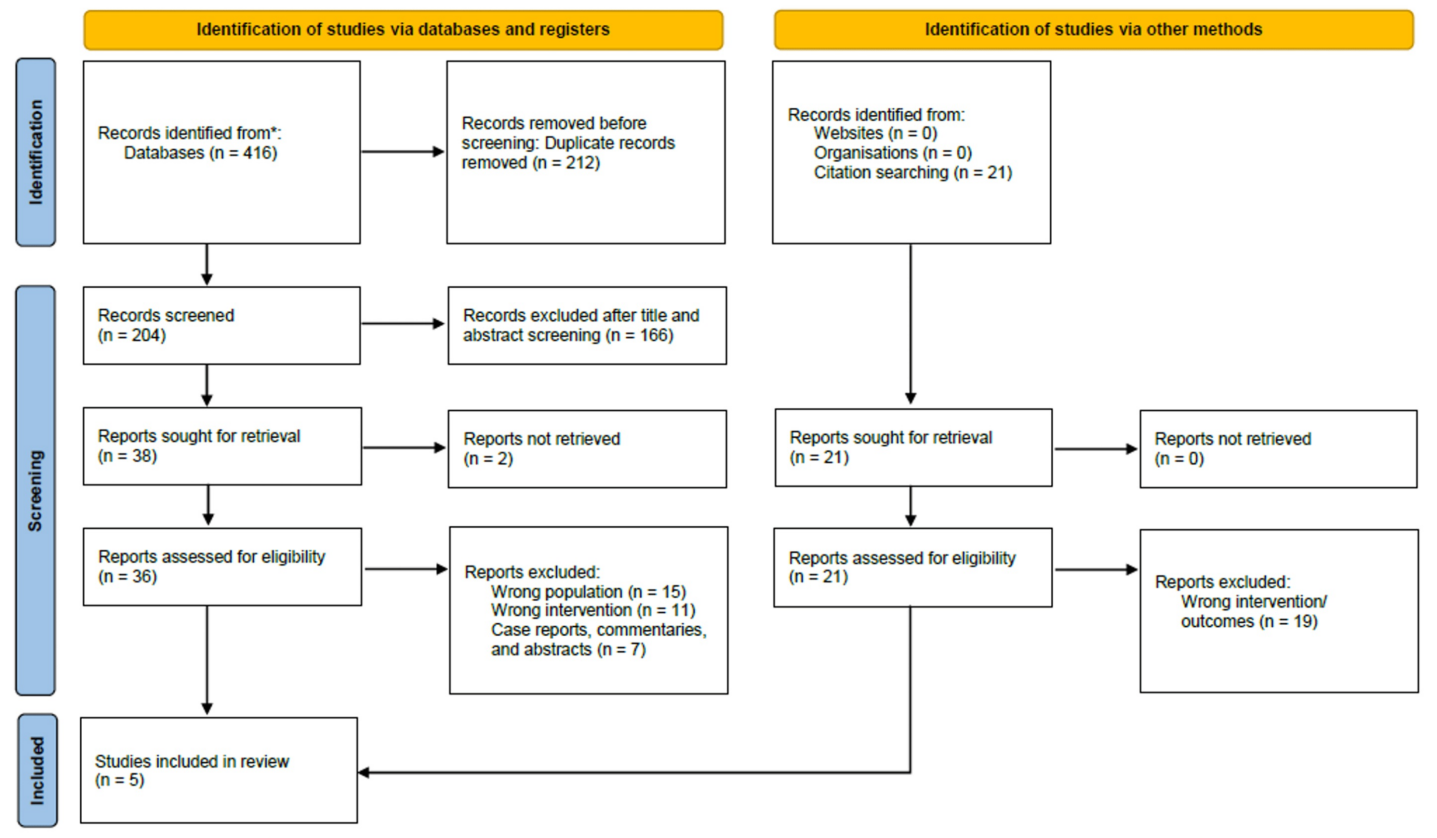
PRISMA flowchart [14] PRISMA: Preferred Reporting Items for Systematic Reviews and Meta-Analyses

The specified search strategy yielded 416 publications (Figure 1). After removing duplicates (n = 212), 204 trials were evaluated based on the title and abstract. Of these, 166 failed to satisfy eligibility criteria, leaving just 38 full-text articles for comprehensive review. Two records were identified through a citation search, and only one was accepted into our review. A total of five satisfied the requirements for eligibility with evidence synthesis for analysis, including three retrospective cohorts, one cross-sectional, and one prospective cohort.

Sociodemographic and Clinical Outcomes

We included five studies with a total of 336 patients, and most of them were males (273, 81.2%). A total of 211 patients were in the laparoscopy group and 125 in the laparotomy group. One study was implemented in the USA [15], one in China [16], one in South Africa [17], one in Turkey [18], and one in Iran [19].

In all the studies, patients in the laparoscopy group remained stable, whereas those in the laparotomy group showed hemodynamic instability [15-19]. This pattern indicates that laparotomy was more commonly required for unstable patients, while less invasive treatments were suitable for those in stable conditions.

The severity of injuries, measured by the Injury Severity Score (ISS), differed across studies. Some provided specific values, while others did not mention them. One study reported a relatively low ISS of 5.3 ± 2.7 for laparoscopy cases and 4.6 ± 2.5 for laparotomy cases [16]. Another study found that laparoscopy cases had an ISS of 6, while laparotomy cases had a much higher ISS of 12.5, highlighting a greater trauma severity in laparotomy patients [17]. However, some studies did not include the ISS data, making direct comparisons difficult [15,18,19].

The type of trauma also varied, but penetrating injuries, such as stab and gunshot wounds, were the most frequent causes. Some studies indicated that stab wounds were more common, with gunshot wounds making up a smaller percentage [15-17]. One study exclusively reported stab wounds in all cases [18], while another did not specify the distribution of trauma types [19]. These findings reflect differences in injury patterns across patient groups and regions.

Main Outcomes

The primary outcomes across the studies demonstrated several advantages of the laparoscopic approach, particularly in stable patients with PAT. Laparoscopy was associated with shorter hospital stays, faster recovery times, less oral intake restriction, and reduced overall healthcare costs [15-19]. One study highlighted the effectiveness of therapeutic laparoscopy in managing abdominal trauma for hemodynamically stable patients, allowing for early discharge and a quicker return to normal activities [16]. Another study echoed these findings, showing no significant differences in outcomes between laparoscopy and laparotomy groups regarding postoperative morbidity but noted the operational and recovery benefits of laparoscopy [17]. However, in cases of retroperitoneal injuries or when patients presented with active bleeding, the laparoscopic approach showed limitations, often necessitating conversion to open surgery. Despite this, laparoscopy remains a strong contender in abdominal trauma management, significantly reducing hospital stay duration, postoperative complications, and overall costs in selected patient populations [18,19].

Complications

Laparoscopy showed a generally lower complication rate compared to laparotomy. For instance, in one study, only 7.7% of the patients undergoing laparoscopy had to convert to open surgery due to complications, such as difficulties managing bleeding or other unforeseen challenges [15]. Another study reported no significant difference in the incidence of serious postoperative morbidities between laparoscopy and laparotomy groups, suggesting that laparoscopy could be equally safe [16]. However, some studies pointed out that retroperitoneal injuries, especially in patients with hemodynamic instability, were difficult to manage laparoscopically, leading to higher conversion rates. This suggests that while laparoscopy is beneficial in selected cases, its limitations become evident in more complex injuries [17,18].

Additionally, one retrospective study found a higher incidence of postoperative ileus and surgical wound infections in the laparotomy group, implying that the invasiveness of open surgery contributed to more severe postoperative complications. In contrast, patients undergoing laparoscopy experienced fewer wound infections and related issues, emphasizing the advantages of the minimally invasive approach (Tables 1, 2) [19].

**Table 1:** Outcome measures of the included studies ISS: Injury Severity Score; NM: not mentioned; LP: laparoscopy; LT: laparotomy

Study ID	Country	Study design	Sociodemographic	Hemodynamic status		Trauma type		Mean ISS		Conversion to open	Complications	Main outcomes
				LP	LT	LP	LT	LP	LT			
Gómez et al., 2022 [15]	USA	Cross-sectional	LP cases = 26; LT cases = 26; mean age: 28; males: 40 (76.9%)	Stable	Unstable	Stab (76.9%) and Gunshot (23.1%)	Stab (76.9%) and Gunshot (23.1%)	NM	NM	2 (7.7%)	The complication rate was lower in the laparoscopic group.	The group receiving total therapeutic laparoscopy had shorter hospital stays, shorter operating times, and less oral intake
Gao et al., 2020 [16]	China	Retrospective cohort	LP cases = 54; LT cases = 53; mean age: 39; males: 83 (77.6%)	Stable	Unstable	Stab and gunshot wounds	Stab and gunshot wounds	5.3 ± 2.7	4.6 ± 2.5	4 (7.4%)	There was no significant difference in the incidence of postoperative serious morbidities between the LP and LT groups	Individuals with PAT whose hemodynamic status is stable may benefit from therapeutic laparoscopy, which could be safe and effective
Koto et al., 2019 [17]	South Africa	Retrospective cohort	LP cases = 45; LT cases = 11; mean age: 31; males: 49 (87.5%)	Stable	Unstable	Stab (91%) and gunshot (62%)	Stab (9%) and gunshot (38%)	6	12.5	11 (19.6%)	Eight patients, or 73% of the total, had active bleeding as their primary reason for conversion	For patients with PAT who are hemodynamically stable, laparoscopic therapy of retroperitoneal injuries is both safe and practicable
Yücel et al., 2017 [18]	Turkey	Prospective cohort	LP cases = 68; LT cases = 13; mean age: 27.5; males: 75 (92.6%)	Stable	Unstable	Stab wound (100%)	Stab wound (100%)	NM	NM	2 (2.9%)	The incidence of death was 4.9%. A 42-month follow-up on average showed no problems, recurrence, or morbidity	Before the patient is released from the hospital, diagnostic laparoscopy should be offered as a safe and effective way to assess the diaphragm for any damage
Shams & Elyasi, 2021 [19]	Iran	Retrospective cohort	LP cases = 18; LT cases = 22; mean age: 33.4; males: 26 (65%)	Stable	Unstable	NM	NM	NM	NM	NM	Patients in the laparotomy group experienced a greater incidence of postoperative ileus and the highest prevalence of surgical wound infection	When used to treat patients with stable hemodynamics and penetrating abdominal trauma, the diagnostic laparoscopic approach dramatically lowers complications, expenses, and hospital stays

**Table 2:** Using ROBINS-I to quantify bias risk ROBINS-I: Risk Of Bias In Nonrandomized Studies-of Interventions; Mod: moderate

Study ID	Bias induced by confusion	Bias in the way participants were chosen	Prejudice in the way interventions are categorized	Bias resulting from departures from the planned interval	Bias resulting from absent data	Bias in the way results are measured	Prejudice in the chosen reported outcome	General prejudice
Gómez et al., 2022 [15]	Mod	Mod	Low	Low	Low	Mod	Mod	Mod
Gao et al., 2020 [16]	Mod	Mod	Low	Low	Low	Mod	Low	Mod
Koto et al., 2019 [17]	Mod	Mod	Mod	Low	Low	Low	Low	Mod
Yücel et al., 2017 [18]	Mod	Mod	Low	Low	Low	Low	Mod	Mod
Shams & Elyasi, 2021 [19]	Low	Low	Low	Low	Low	Mod	Low	Low

Discussion

Our systematic evaluation revealed several benefits of the laparoscopic method, especially in stable patients with penetrating abdominal injuries. Laparoscopy was associated with shorter hospital stays, faster recovery times, less oral intake restriction, and reduced overall healthcare costs [15-19]. This result aligns with Li et al., who found that when hemodynamically stable patients with PAT undergo laparoscopy, the procedure's perioperative results are effectively improved and its consequences are minimized. This finding should be made more widely known in therapeutic settings [20]. Additionally, O’Malley et al. found that in a certain patient subgroup, laparoscopy may significantly impact PAT; however, surgeon experience is also a key factor [11]. Laparoscopy is a diagnostic, therapeutic, and screening method that is particularly useful when there is a possibility of diaphragm damage. It is sensitive in diagnosing when a laparotomy is required but less reliable in identifying hollow visceral injuries. When utilized in institutions with the appropriate training, it could be a therapeutic aid [20]. 

However, we found that in cases of retroperitoneal injuries or when patients presented with active bleeding, the laparoscopic approach showed limitations, often necessitating conversion to open surgery [17]. Wang et al. found that for some patients, laparoscopic surgery is a more sensible option than laparotomy. Nevertheless, the surgeon's experience and the available resources should be considered when deciding whether to undergo a laparoscopy [21].

In this review, laparoscopy showed a generally lower complication rate compared to laparotomy. For instance, in one study, only 7.7% of the patients undergoing laparoscopy had to convert to open surgery due to difficulty in managing bleeding or other unforeseen challenges [15-18]. Patients undergoing laparoscopy also experienced fewer wound infections and related issues, emphasizing the advantages of this minimally invasive approach [19].

Moreover, postoperative complications and the perioperative mortality rate were significantly lower in the laparoscopy group than in the laparotomy group. The two most frequent side effects of open laparotomy and laparoscopy included wound infection and pneumonia. A meta-analysis of the case series studies showed that the pooled incidence of problems for laparoscopic patients was 0.08. Hence, laparoscopy may lower the incidence of postoperative complications compared to laparotomy, but it may not entirely prevent it. This finding might act as a motivation to keep improving to maintain the lowest possible rate of problems. Additionally, the surgeon must have experience performing laparoscopic surgery to minimize the rate of problems; they should also be able to diagnose patients quickly and manage challenges appropriately [20].

According to the results, individuals with penetrating abdominal injuries who are hemodynamically stable should be managed with laparoscopy as a first-line treatment option due to shorter hospital stays, fewer surgical problems, and lower medical expenses. However, caution is advised in patients with complex injuries, such as retroperitoneal trauma or uncontrolled bleeding, where laparotomy may be necessary. Laparoscopy could also reduce the burden of open surgery, potentially improving patient outcomes and optimizing hospital resource utilization.

Strengths and limitations

This review consolidated the information from several trials comparing laparoscopy with laparotomy in the event of penetrating abdominal injuries, providing a broad perspective on their respective outcomes. Data repeatedly demonstrated that laparoscopy is a secure and useful substitute, especially in selected patient populations. The focus on clinically meaningful outcomes, such as complication rates and conversion rates, makes the results directly applicable to surgical practice.

One limitation is the variability in study design, with both retrospective and prospective studies included, which may introduce biases in data interpretation. Additionally, most studies focused on stable patients, limiting the generalizability of the findings to severe trauma cases. Conversion rates were higher in patients with retroperitoneal or complex injuries, indicating that laparoscopy might not be suitable for all types of PAT. To improve the body of data, further randomized controlled studies are required.

## Conclusions

When treating penetrating abdominal injuries, laparoscopy is less intrusive, enables faster recovery, and produces fewer postoperative problems than laparotomy, especially for stable patients. However, its limitations in managing more complex injuries warrant careful patient selection and readiness to convert to open surgery when necessary. Further investigation, especially randomized studies, is required to confirm laparoscopy's place in this sector.
